# Detection of Rare Methyl-CpG Binding Protein 2 Gene Missense Mutations in Patients With Schizophrenia

**DOI:** 10.3389/fgene.2020.00476

**Published:** 2020-05-08

**Authors:** Chia-Hsiang Chen, Min-Chih Cheng, Ailing Huang, Tsung-Ming Hu, Lieh-Yung Ping, Yu-Syuan Chang

**Affiliations:** ^1^Department of Psychiatry, Chang Gung Memorial Hospital-Linkou, Taoyuan, Taiwan; ^2^Department and Graduate Institute of Biomedical Sciences, Chang Gung University, Taoyuan, Taiwan; ^3^Department of Psychiatry, Yuli Mental Health Research Center, Yuli Branch, Taipei Veterans General Hospital, Hualien, Taiwan

**Keywords:** schizophrenia, Rett syndrome, MECP2, mutation, heterogeneity

## Abstract

Deleterious mutations of MECP2 are responsible for Rett syndrome, a severe X-linked childhood neurodevelopmental disorder predominates in females, male patients are considered fatal. However, increasing reports indicate that some MECP2 mutations may also present various neuropsychiatric phenotypes, including intellectual disability, autism spectrum disorder, depression, cocaine addiction, and schizophrenia in both males and females, suggesting varied clinical expressivity in some MECP2 mutations. Most of the MECP2 mutations are private *de novo* mutations. To understand whether MECP2 mutations are associated with schizophrenia, we systematically screen for mutations at the protein-coding regions of the MECP2 gene in a sample of 404 schizophrenic patients (171 females, 233 males) and 390 non-psychotic controls (171 females, 218 males). We identified six rare missense mutations in this sample, including T197M in one male patient and two female controls, L201V in nine patients (three males and six females) and 4 controls (three females and one male), L213V in one female patient, A358T in one male patient and one female control, P376S in one female patient, and P419S in one male patient. These mutations had been reported to be present in patients with various neuropsychiatric disorders other than Rett syndrome in the literature. Furthermore, we detected a novel double-missense mutation P376S-P419R in a male patient. The family study revealed that his affected sister also had this mutation. The mutation was transmitted from their mother who had a mild cognitive deficit. Our findings suggest that rare MECP2 mutations exist in some schizophrenia patients and the MECP2 gene could be considered a risk gene of schizophrenia.

## Introduction

Deleterious mutations of the methyl-CpG binding protein 2 (MECP2) gene are well-known to cause Rett syndrome, a severe X-linked neurodevelopmental disorder in childhood characterized by progressive neurological deficits and psychomotor retardation after a brief normal development (Ip et al., 2018). Rett syndrome is predominant in females because male patients with similar mutations are considered fatal. There are more than 900 different mutations that scatter throughout the entire MECP2 gene in the databases of Rett syndrome and MECP2 (Townend et al., 2018). Most of the MECP2 mutations are private *de novo* mutations. However, there is an increasing appreciation that the clinical presentations of MECP2 mutations are not limited to typical Rett syndrome. Some MECP2 mutations may present a wide range of neuropsychiatric conditions including autism spectrum (Qiu, 2018), intellectual disability (Berger-Sweeney, 2011; Bassani et al., 2013), schizophrenia (Sweatt and Tamminga, 2016), depression, and cocaine addiction (Ausio, 2016). These neuropsychiatric manifestations were found in both male and female patients, indicating MECP2 mutations have a high level of clinical heterogeneity.

Schizophrenia is a complex mental disorder with genetic factors playing a major role in its causes. The estimated heritability of schizophrenia is around 80–85% (Cardno and Gottesman, 2000). The current understanding of the genetic basis of schizophrenia includes common variants with small clinical effects and rare variants with moderate to large clinical effects (Avramopoulos, 2018). More than one hundred common variants with small effect sizes have been reported to be associated with schizophrenia from the study of the Psychiatric Genomic Consortium (Schizophrenia Working Group of the Psychiatric Genomics Consrotium, 2014). Additionally, numerous rare genetic variants with moderate to large effect sizes such as chromosomal abnormalities, copy number variations, small insertion and deletion, and nucleotide changes were found to be associated with schizophrenia (Foley et al., 2017; Avramopoulos, 2018). These results indicate that the genetic underpinnings of schizophrenia are very complex, heterogeneous, and highly individualized. Hence, accurate molecular genetic diagnosis of each affected patient is essential for genetic counseling, the guidance of clinical management, and investigation of the pathogenesis of patients with schizophrenia (Fraguas et al., 2017; Sullivan et al., 2018).

As part of the molecular genetic study series of schizophrenia, we were interested to understand whether rare genetic mutations of MECP2 are present in patients with schizophrenia. To address this issue, we conducted a systematic screening for mutations at the protein-coding regions of the MECP2 gene in a sample of schizophrenia and control subjects from Taiwan. Here, we report our findings in this communication.

## Materials and Methods

### Subjects

All the subjects were Han Chinese residing in Taiwan. Patients meeting the diagnostic criteria for chronic schizophrenia according to the Diagnostic and Statistical Manual of Mental Disorders, Fourth Edition, text revision (DSM-IV-TR; American Psychiatric Association (APA), 2000) were recruited from the Department of Psychiatry, Yuli Mental Health Research Center, Yuli Branch, Taipei Veterans General Hospital, Hualien, Taiwan. Non-psychotic controls were recruited from a medical center’s Department of Family Medicine in eastern Taiwan. The study was approved by the Institutional Review Board of Antai-Tian-Sheng Memorial Hospital with the approval number of 101020, and by the Institutional Review Board of Chang Gung Memorial Hospital-Linkou, Taoyuan City, Taiwan, with the approval number of 105–6162C. Written informed consent was obtained from each subject after a full explanation of this study. A total of 404 schizophrenic patients (171 females, 233 males) and 390 non-psychotic controls (171 females, 218 males) were screened for MECP2 mutations in this study. Genomic DNA was extracted from venous blood using the Gentra Genomic DNA Purification kit (Gentra Systems, Inc., Minneapolis, MN, United States) according to the manufacturer’s instructions. For the family study of the patient with a double-missense mutation of MECP2, written informed consent was obtained from each family member who agreed to participate in this study except one who refused to participate. We also obtained written informed consent from the family for the publication of clinical data and indirectly identifiable information in this study.

### MECP2 Mutation Screening by Sanger Sequencing

The MECP2 gene is located at X chromosome q28 and has four protein-coding exons that generate two isoforms of MECP2 protein by alternative splicing (Mnatzakanian et al., 2004). We designed five pairs of primers to PCR amplify four protein-coding exons of the MECP2 gene. The primer sequences, optimal annealing temperature, and size of amplicon are listed in Table 1. In brief, PCR amplification was performed in 15 μl reaction mixtures containing genomic DNA (50 ng), 1 μM each of forward and reverse primer, 0.2 mM of dNTP, 1X PCR buffer and 0.75 unit of Taq polymerase. The PCR conditions included initial denaturation at 95°C for 5 min, followed by 30 cycles of 95°C for 1 min, optimal annealing temperature for each amplicon for 1 min, and elongation at 72°C for 1 min. After PCR, aliquots of amplicon were cleaned by the Illustra^TM^ ExoProStar^TM^ 1-Step Kit (GE Healthcare Bio-Sciences Corp., NJ, United States), then subjected to sequencing reaction using an ABI Prism^TM^ BigDye^TM^ Terminator Cycle Sequencing Ready Reaction Kit Version 3.1 and implemented by the ABI autosequencer 3730 (Perkin Elmer Applied Biosystem, Foster City, CA, United States). The authenticity of mutations identified was verified by repeated PCR and sequencing in forward and reverse directions.

**TABLE 1:** Primer sequences and PCR conditions for mutation screening of MECP2 gene.

Primer	Forward	Reverse	Size (bp)	Ta (°C)
E1	5′-ccgaaatggacaggaaatct-3′	5′-agggggagggtagagaggag-3′	468 bp	60°C
E2	5′-acgtgccagtaatttgcagc-3′	5′-ggcgaccaagtaaccctaca-3′	521 bp	65°C
E3	5′-ttctgcagactggcatgttc-3′	5′-tagagggcctgctaccttga-3′	603 bp	60°C
E4.1	5′-tgccctatctctgacattgct-3′	5′-cacttccttcacctcgatgc-3′	649 bp	65°C
E4.2	5′-aaagaaagccgtgaaggagctc-3′	5′-attcttgttggtttgctttgc-3′	649 bp	60°C

*Ta: annealing temperature.*

### Verification of the MECP2 Double-Missense Mutation

To conduct the family study with the double-missense mutation, a specific amplicon of 373 bp was obtained using the primer pair of MECP2-F1 (5′-GCCTTG GCATGGAGGATGAAACA-3′) and MECP2-R1 (5′-AAAAGCAAGGAG AGCAGCCCCAA-3′) from each available DNA. The PCR conditions consisted of an initial denaturation at 95°C for 5 min, followed by 30 cycles of 95°C for 30 s, annealing at 95°C for 30 s, and elongation at 72°C for 30 s. An aliquot of the amplicon was subjected to Sanger sequencing using the MECP2-F1 as the sequencing primer.

### Whole Exome Sequencing and Variant Analysis

Whole exome sequencing analysis was performed in the family with the MECP2 double-missense mutation to explore whether there are other mutations associated with schizophrenia in this family. The exome library was prepared using the Illumina TruSeq Exome Library Prep Kit (Illumina). Paired-end whole-exome sequencing was performed using the Illumina HighSeq2000 platform at Yourgene Bioscience, Taiwan^1^. After quality control, raw sequencing reads were aligned to reference genome hg19. SAMtools and Genome Analysis Toolkit (GATK) were further used to refine the local alignment and to generate variant calling file (VCF) for each subject sequenced. Variants were annotated and analyzed using SeqsLab software (Atgenomix, Taipei, Taiwan) to search for mutations segregating with schizophrenia. The annotated VCF data of the family were analyzed under different inheritance modes, including autosomal dominant, autosomal recessive, X-linked and *de novo*.

### Bioinformatics Analysis

The allele frequencies of MECP2 missense mutations identified in this study were checked in the SNP website^2^ and Taiwan BioBank^3^. The potential functional consequences of missense mutations identified in this study were assessed using Polyphen-2^4^ and SIFT^5^.

## Results

### Detection of Six Missense Mutations of MECP2

**FIGURE 1:**
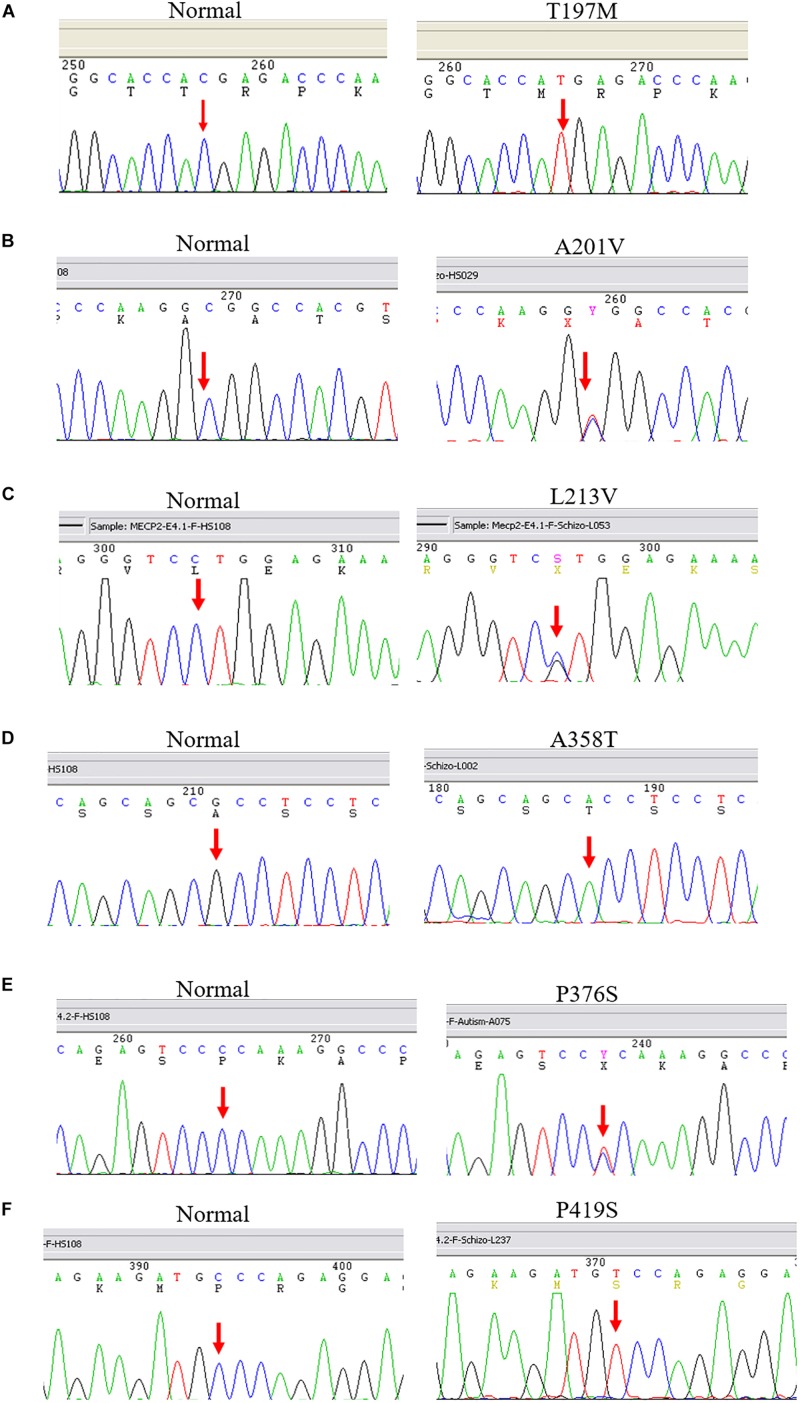
Representative results of Sanger sequencing of six missense mutations identified in this study. The left panel is the reference sequence, the right panel is the mutant sequences. **(A)** A C-to-T substitution at the MECP2 cDNA sequence 590 changes amino acid 197 from threonine to methionine in a male patient. **(B)** A C-to-T change at the MECP2 cDNA sequence 602 leads to an amino acid alteration from alanine to valine at sequence 201 in a female patient. **(C)** A C-to-G substitution at the MECP2 cDNA sequence 637 results in amino acid sequence change from leucine to valine at position 213 in a female patient. **(D)** A G-to-A substitution at the MECP2 cDNA sequence 1072 changes amino acid sequence 358 from alanine to threonine in a female patient. **(E)** A C-to-T aleration at the MECP2 cDNA sequence 1126 changes the amino acid sequence 376 from proline to serine in a female patient. **(F)** A C-to-T change at the MECP2 cDNA sequence 1255 results in the alertation of amino acid sequence 419 from proline to serine in a male patient.

After screening 404 schizophrenic patients (171 females, 233 males) and 390 non-psychotic controls (171 females, 218 males), we identified six MECP2 missense mutations in this sample, including T197M in one male patient and two female controls, L201V in nine patients (three males and six females) and 4 controls (three females and one male), L213V in one female patient, A358T in one male patient and one female control, P376S in one female patient, and P419S in one male patient. The findings are summarized in Table 2. The representative results of Sanger sequencing of these missense mutations are shown in Figure 1.

**TABLE 2:** Rare missense mutations identified in this study.

Missense mutation	Nucleotide change at cDNA position	Change of amino acid and position	Patients (*n* = 404)	Controls (*n* = 390)
1	c.590 C > T	T197M	1 male	2 females
2	c.602C > T	A201V	3 males, 6 females	3 females, 1 male
3	c.637 C > G	L213V	1 female	0
4	c.1072 G > A	A358T	1 male	1 female
5	c.1126 C > T	P376S	1 female	0
6	c.1255 C > T	P419S	1 male	0

### Detection of a Double-Missense Mutation of MECP2 in a Multiplex Family

**FIGURE 2:**
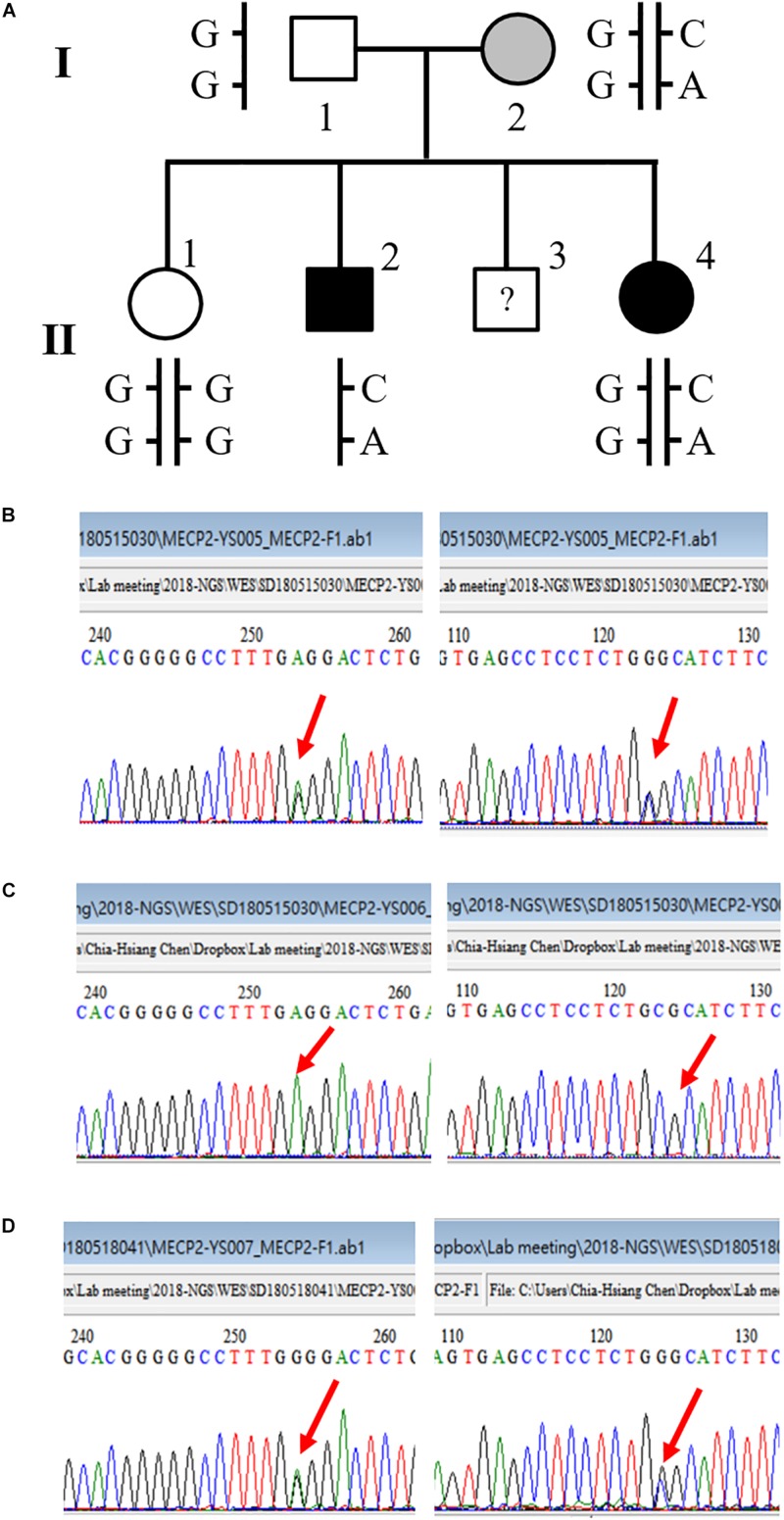
A novel double-missense mutation was identified in a multiplex family. **(A)** Pedigree of the family with two affected siblings. **(B)** Sequencing results of P376S and P419R double mutation of the mother (I-2). **(C)** Sequencing results of P376S and P419R double mutation of the son affected with chronic schizophrenia (II-2). **(D)** Sequencing results of P376S and P419R double mutation of the affected daughter (II-4).

We also detected a double-missense mutation in a male patient. The first mutation was a G-to-A nucleotide substitution that resulted in a change of amino acid sequence from proline to serine at codon 376, designated P376S. The second mutation was a G-to-C substitution that led to a change of amino acid sequence from proline to arginine at codon 419, designated P419R. Tracing the family history of this patient, we found that the patient had a sister who was diagnosed with schizophrenia as well. We then conducted a genetic analysis of this family. The pedigree of this family and the sequencing results are shown in Figure 2. This double-missense mutation was transmitted from the mother who had a mild cognitive deficit. This double missense mutation was not present in the unaffected elder sister (II-1) and the father I-1. To rule out the possibility that other genetic mutations may be associated with schizophrenia in this family, we conducted a whole-exome sequencing analysis for this family. We confirmed the co-segregation of this double-missense mutation with schizophrenia in this family using the whole-exome sequencing data. We did not find any other variants that may associate with schizophrenia in this family using a recessive or X-linked inheritance model.

### Clinical Findings of the Family

Patient II-2 was a male patient in his 60’s. He was diagnosed with schizophrenia when he was in his 20’s. His symptoms began with auditory hallucination, self-talking, unstable emotion, bizarre behavior, an idea of reference, and delusion of persecution. He graduated from high school and had an unremarkable developmental history before the onset of his mental illness. He did not respond well to antipsychotics treatment, so his psychotic symptoms persisted. He also manifested violent behavior and suicide attempts off and on during his illness. Hence, he was repeatedly hospitalized for acute psychiatric wards several times. Later in his 50’s, he suffered from a seizure attack. The examination of computed tomography of the brain did not reveal any abnormalities, but electroencephalography showed abnormal brain activities. Hence, he received anticonvulsants treatment in addition to antipsychotics since then. Finally, he was placed in a chronic psychiatric hospital because of his poor social function and persistence of prominent psychotic symptoms.

Patient II-4 was a female patient in her 50’s. She was diagnosed with schizophrenia when she was in her 20’s. Her symptoms began with auditory hallucination, hot temper, aggressive behavior, and poor sleep. Before the onset of her mental symptoms, she had an unremarkable developmental history. However, her school performance was poor. She managed to graduate from high school, however, she could not maintain a stable job before the onset of her mental illness. After the onset of her mental illness, she was treated with antipsychotics, but her response to antipsychotic medication was not satisfactory. She continued to have self-talking and residual auditory hallucination, delusion, and the idea of reference. Additionally, she has prominent negative symptoms, including blunt affect, poor self-hygiene, social withdrawal, and avolition. She was not hospitalized for acute psychiatric wards during her mental illness, instead, she stayed in the day hospital for rehabilitation for a long time.

Subject II-1 was a woman in her 60’s. She is the main caregiver of this family. She had a normal developmental history and social function. The father (I-1) was in his 90’s who also did not have any history of mental illness. The mother (I-2) was in her 80’s. She did not have any history of mental illness before, nor did she meet any formal diagnosis of mental illness during this study. She did not have any formal jobs after graduation from elementary school. Also, according to the description of her daughter (II-1), she can only do some simple housekeeping work and has a rigid personality trait. It seems that she has a mild cognitive deficit, but she did not receive any formal psychometric assessment. Subject II-3 refused to join the study.

### Bioinformatics Analysis of Missense Mutations Identified in This Study

The results of the bioinformatics analysis are summarized in Table 3. All the missense mutations identified in this study were predicted to be non-pathogenic using two computer programs.

**TABLE 3:** Allele frequency and functional prediction of MECP2 missense mutations identified in this study.

Mutation at cDNA position	Mutation at protein position	dbSNP No.	Taiwan Biobank	GMAF	ExAC	SIFT Score	PolyPhen2 Score
c.590 C > T	T197M	rs61749714	0.001745	0.0003	0.00055	0.12 Tolerated	0.008 Benign
c.602 C > T	A201V	rs61748381	0.01	0.0048	0.00154	0.21 Tolerated	0.002 Benign
c.637 C > G	L213V	rs782473355	0.0003		0.00001	1 Tolerated	0.001 Benign
c.1072 G > A	A358T	rs147017239	0.0009	0.0003	0.00027	0.75 Tolerated	0.000 Benign
c.1126 C > T	P376S	rs61752387	0.0048	0.0003	0.00086	0.11 Tolerated	0.000 Benign
c.1255 C > T	P419S	rs140258520	0		0.00005	0.17 Tolerated	0.025 Benign
c.1255 C > G	P419R		0			0.3 Tolerated	0.025 Benign

*GMAF, Global minor allele frequency. The Exome Aggregation Consortium (ExAC).*

## Discussion

In this study, we detected a total of 6 rare single missense mutations and a novel double-missense mutation of the MECP2 gene after screening 404 patients and 390 control subjects. To our knowledge, there were only two double mutations of MECP2 (G185V-R255X and P179S-R255X) reported in two patients with Rett syndrome, respectively (Kharrat et al., 2017). Notably, the double missense mutation (P376S-P419R) identified in our study is a novel one that was not reported in the literature to our knowledge.

The P376S mutation was detected in another female patient with schizophrenia in this study (Table 2). In the literature, the P376S mutation had been reported in a female with Rett syndrome and her unaffected father (Laccone et al., 2002), a female with classical Rett syndrome (Zhao et al., 2014), a male with intellectual disability, a girl with atypical Rett syndrome and her unaffected father (Bourdon et al., 2003). Together, these results suggest that this mutation has varied clinical expressivity. Our bioinformatic analysis showed that the P376S mutation is a benign one, however, in a recent study, the P376S was detected in a male ASD patient and functional study of this mutation revealed that overexpression of the P376S mutation led to a loss of inhibition of dendritic spine growth and enhanced axonal growth when compared to the wild-type, suggesting the P376S mutation might be pathogenic (Wen et al., 2017).

The P419R mutation identified in our study is a novel one, not reported in the databases of Rett syndrome, population databases, and in our control sample. At the same position, we detected another missense mutation P419S in a male patient with schizophrenia. This mutation was not detected in our 390 control samples, nor in Taiwan Biobank, but this mutation was reported in the Exome Aggregation Consortium (ExAc) with the allele frequency of 0.00005 (Table 3), and in a girl with Rett syndrome (Gauthier et al., 2005). Further studies are needed to characterize the functional significance of both P419S and P419R mutations, given they were predicted benign in our bioinformatics analysis.

We also detected four other missense mutations in this study, i.e., T197M, A201V, L213V, and A358T (Table 2). These missense mutations had been reported in the literature with various clinical manifestations. The T197M mutation was reported in a male with congenital encephalopathy, microcephaly, and severe developmental (Laccone et al., 2002), a boy with non-progressive encephalopathy, microcephaly, autistic behavior, and profound speech deficit (Bourdon et al., 2003), and a male with autism (Wang et al., 2013). In this study, the T197M was detected in two female control subjects and one male patient with schizophrenia. The absence of clinical phenotypes of these two female carriers might be due to the inactivation of the mutant X chromosome, but they might transmit this mutation to their offspring.

The A201V variant was detected in nine patients (3 males and 6 females) and four in controls (3 females and 1 male). This mutation also had heterogeneous clinical presentations. Lam and colleagues reported on this mutation in a Chinese female patient with autism and mental retardation. They also detected this mutation in unaffected males like our findings in this study. Hence, they considered this mutation as a neutral polymorphism (Lam et al., 2000). The A201V mutation was reported in another Chinese male patient with autism in another study. This mutation was transmitted from his unaffected mother. His grandmother was also a carrier of this mutation who had depressive symptoms (Wang et al., 2013). The A201V was also reported in one Korean patient with Rett syndrome but none in more than 100 controls, hence, the authors considered this mutation as apparently pathogenic in their report (Kim et al., 2006). The A201V was detected in three patients after sequencing 219 Japanese patients with typical or atypical Rett syndrome (Fukuda et al., 2005) and in one out of 118 Japanese patients with mental retardation (Ylisaukko-Oja et al., 2005). However, the allele frequency in the Japanese population was 1.47 (Fukuda et al., 2005), suggesting the clinical effect of this mutation might be small or moderate. The allele frequency of this mutation in Taiwan BioBank is 1%, similar to that in the Japanese population. Nevertheless, the allele frequencies of this mutation in the database of the Global minor allele frequency (GMAF) and The Exome Aggregation Consortium (ExAC) are very rare (Table 3). Thus, it is likely that the varied clinical presentations of this mutation may be influenced by other factors.

The L213V mutation was found in one female patient with schizophrenia, but none in control subjects in this study. The allele frequency of this allele is very rare in Taiwan Biobank (0.0003) and ExAc (0.00001) (Table 3). The further functional study is needed to address the pathogenicity of this mutation.

The A358T missense mutation was found in one male schizophrenia patient and one female control subject in our study. The frequency of this allele is very rare in Taiwan Biobank and several population databases (Table 3). This mutation was reported in one female patient with Rett syndrome (Li et al., 2007), one male patient with epileptic encephalopathy (Wong and Li, 2007), and one female patient with intellectual disability (Zvereff et al., 2012). But this mutation was also detected in an unaffected father (Zvereff et al., 2012), suggesting the varied expressivity of this mutation.

Together, these missense mutations were present not only in patients with various neurodevelopmental disorders but also in unaffected subjects. This might be due to several factors. First, the pathogenic effect of these mutations is not as severe as those causing typical Rett syndrome. This point was supported by the silico analysis showing that all these missense mutations are not pathogenic. Hence, male carriers of these mutations are not fatal and both male and female carriers could express various behavioral symptoms other than Rett syndrome. Second, other genetic or non-genetic factors might modify or even protect the pathogenesis of these mutations. Hence carriers of these mutations had a varied expression of phenotype from normal to various neuropsychiatric diagnoses. Third, normal female carriers of these mutations could also be attributed to the random inactivation effect of the X chromosome, but they could transmit these mutations to their offspring who may express clinical phenotypes.

Several studies have reported schizophrenia is one of the varied clinical expressivities of MECP2 mutation. For example, an A140V mutation in a boy presented with developmental receptive language disorder and childhood-onset schizophrenia (Cohen et al., 2002), a T196S mutation in a female with schizophrenia (Shibayama et al., 2004), and an R202C mutation in a female patient with schizophrenia (McCarthy et al., 2014). Our findings add further evidence to support that MECP2 missense mutations exist in some patients with the clinical diagnosis of schizophrenia. Thus, the MECP2 gene shall be considered as a susceptible gene of schizophrenia.

The varied clinical expressivity of some MECP2 mutations could be attributed to several unique characteristics of the MECP2 protein. First, MECP2 protein is abundant in the central nervous system and is widely distributed throughout the whole genome like histones (Skene et al., 2010). The MECP2 protein interacts with a variety of partner proteins and binds both methylated and non-methylated DNA sequences. Thus, the MECP2 gene can regulate the expression of multiple genes in the brain (Cohen and Greenberg, 2010; Skene et al., 2010). Second, MECP2 protein is also involved in the splicing of mRNA and the biogenesis of microRNA in the brain (Woo and Kim, 2014; Ip et al., 2018), which in turn modulates multiple genes involved in early human neurogenesis, brain maturation, and development (Mellios et al., 2018). Third, MECP2 is subjected to various post-translational modifications, including phosphorylation, acetylation, ubiquitination, and sumoylation, which may fine-tune its regulation of gene expression involved in neural differentiation and synaptic plasticity (Bellini et al., 2014; Zhong et al., 2018). Fourth, MECP2 protein belongs to the family of intrinsically disordered protein which was defined as a lack of stable secondary or tertiary structure under physiological conditions (Uversky et al., 2008; Hansen et al., 2010). The proper function of the intrinsically disordered protein is dependent on its conformation that is influenced by local conditions (Uversky et al., 2008). Hence, MECP2 mutations may have various conformations of as defined by the genetic and non-genetic factors of each patient.

In summary, we suggest that while serious deleterious mutations of the MECP2 gene are responsible for the severe clinical phenotypes of Rett syndrome in female patients and lethal in male patients, some less serious missense mutations may result in other neuropsychiatric manifestations given the versatile function of MECP2 in the brain. In this study, we demonstrated that schizophrenia is one of the varied clinical presentations of MECP2 mutations and MECP2 can be considered a risk gene of schizophrenia. Our findings shall contribute to our understanding of the molecular genetics of schizophrenia.

## Data Availability Statement

The datasets for this article are not publicly available due to concerns regarding participant/patient anonymity. Requests to access the datasets should be directed to the corresponding author.

## Ethics Statement

The studies involving human participants were reviewed and approved by Institutional Review Board of Antai-Tian-Sheng Memorial Hospital with the approval number of 101020, and by the Institutional Review Board of Chang Gung Memorial Hospital-Linkou, Taoyuan City, Taiwan, with the approval number of 105-6162C. The patients/participants provided their written informed consent to participate in this study. Written informed consent was obtained from the individual(s) for the publication of any potentially identifiable images or data included in this article.

## Author Contributions

C-HC designed the study and obtained research funding. C-HC, AH, M-CC, T-MH, and L-YP diagnosed and recruited the study samples. Y-SC conducted the experiments. C-HC and MC-C analyzed and interpreted the data. C-HC wrote the first draft of the manuscript. All authors contributed to and have approved the final manuscript.

## Conflict of Interest

The authors declare that the research was conducted in the absence of any commercial or financial relationships that could be construed as a potential conflict of interest.

## Footnotes

1 www.yourgene.com.tw

2 https://www.ncbi.nlm.nih.gov/snp/

3 https://taiwanview.twbiobank.org.tw/index

4 http://genetics.bwh.harvard.edu/pph2/

5 http://sift.jcvi.org/
